# The Surgical Treatment of Dilated Stomach

**Published:** 1902-12-20

**Authors:** Leonard A. Bidwell

**Affiliations:** Surgeon to the Hospital, Dean of the Postgraduate College


### Hospital Clinics and Medical Progress.

### THE SURGICAL TREATMENT OF DILATED STOMACH.

A Postgraduate Lecture delivered at the West London Hospital. By Leonard A. Bidwell, F.R.C.S., Surgeon to the Hospital, Dean of the Postgraduate College.

To my mind the existence of dilatation of the stomach is in itself a sufficient indication for operative treatment, especially if there be the least suspicion that the condition is due to malignant disease. Even in cases due to cicatricial stenosis, the state of a patient is one of great misery, and the longer the condition is left untreated the worse the patient's general state becomes. As will be stated later, the prognosis is so favourable in non-malignant cases that we need not hesitate on that score, and should the case prove to be malignant the only time at which a radical operation can be performed is at a stage when an accurate diagnosis is impossible. These considerations lead me to urge an exploratory operation in every case of dilated stomach which has existed for any length of time irrespective of other symptoms. Of course the operation is not merely exploratory, since some more radical action will be taken when the exact nature of the disease has been determined after opening the abdomen. The exact operation will depend a good deal upon whether the case is malignant or non-malignant, and in the former case upon the extent of the disease.

In cases of malignant disease the ideal operation, of course, is pylorectomy; but this is a serious operation, especially on patients who have become exhausted by want of food. I therefore prefer, in all cases, to perform a gastro-enterostomy, and when the pylorus is sufficiently movable to allow of its removal to perform pylorectomy after an interval of two or three weeks, during which time the patient is fed up and his general health improved. Practically no other operation need be considered in cases of malignant disease. I have performed pyloroplasty, but should not recommend it again.

With regard to gastro-enterostomy there is a good deal of difference of opinion as to the relative merits of the anterior and posterior operations. It is claimed that the opening in a posterior gastro-enterostomy will afford a better passage to the stomach contents; but this is naturally only the case when the patient is lying down, and is not a fact when the patient stands, since the opening is at a fixed point owing to the hole in the meso-colon. On the other hand, after an anterior gastro-enterostomy, the weight of the jejunum will pull on the stomach and tend to make the anastomatic opening the lowest point of the stomach. Personally I am very well satisfied with the results of both methods, and I can hardly say which I think is the better.

The only other operative treatment of malignant disease of the pylorus is that suggested by Bernays, who opened the stomach and curetted the growth thoroughly, thus re-establishing the lumen; this, however, can only be palliative, and is probably a rather dangerous proceeding. In cases of malignant disease of the stomach, in which the pyloric orifice is not obstructed, a certain amount of comfort and prolongation of life can be obtained by performing a gastro-enterostomy on the proximal side of the growth.

In dilatation of the stomach from cicatricial contraction, gastro-enterostomy will, of course, give relief, and is practically always a suitable operation; in cases, however, where the anterior wall of the pylorus is free from cicatricial tissue the operation of pyloroplasty may be performed. This operation is, however, open to the objection that the pyloric orifice is no longer the lowest point of the stomach except in cases where the pylorus is very movable, and I now reserve its employment for these cases. The same may be said of Loretta's operation, or digital dilatation; the operation, too, is open to the objection that a recurrence of the stenosis may occur; in some cases, however, the cure is permanent. As a modification of this operation, in cases of recent dilatation, more especially when due to spasm, dilatation of the pylorus can be effected by invagination of the stomach wall through the pylorus with the finger without opening the stomach.

In some cases where the dilatation is of old standing, it is found that it does not subside after gastro-enterostomy, and in such cases the operation of gastro-plication, or the formation of pleats in the stomach, is recommended. I need only say that this operation should never be performed as a substitute for, but only as an accessory to, gastro-enterostomy. Lastly, in cases of dilatation dependent on adhesions about the pylorus, the division of these adhesions (gastrolysis) is indicated.

I do not intend to enter into any details as to the performance of these operations; suffice it to say that I have never employed any buttons, bobbins, or other mechanical appliances, but have relied entirely on simple sutures, which are always passed through the sub mucous as well as the serous and muscular coats.

I will now pass on to the prognosis and results of these operations. In the first place, the prognosis in non-malignant cases is almost invariably good, not only as regards the immediate but as regards the remote results also. I find that I have performed 18 operations for nonmalignant stricture, all of which patients are alive and well, and in only one case was there a recurrence of dilatation.

In malignant disease, on the other hand, the results are by no means so satisfactory; in fact, the immediate mortality is high, owing no doubt to the anæmia and general debility produced by the malignant growth; thus out of 16 cases, 6 died within 14 days of the operation, while several others died within 6 months, and two years is the longest period which any lived. In cases of pylorectomy recurrence occurred in two cases at the end of a year. Subjoined is a rough analysis of my 34 operations for dilated stomach:—

Total number of cases 34, with 6 deaths; mortality 17·6 per cent. Of these malignant cases 16, with 6 deaths; mortality 37·5 per cent. Non-malignant cases 18 with no death.

With regard to the nature of the operation:—

There were 17 gastro-enterostomies with 4 deaths; mortality 23 5 per cent.; but of these 11 were done for malignant disease with 4 deaths, or 36·3 per cent., and 6 were for non-malignant stricture with no death.

There were 7 cases of pyloroplasty with 1 death, or 14·2 per cent. mortality; but of these 1 case was for malignant disease and died; and 6 cases were for non-malignant stricture with no death.

There were 4 cases, of digital dilatation of the pylorus, all for non-malignant disease with no death.

There were 2 cases of gastrolysis (division of adhesions) for non-malignant disease with no death.

There were 4 cases of pylorectomy for malignant disease with 1 death, or 25 per cent. mortality.

Most of the fatal cases were advanced and neglected ones, and I have every confidence that had they been operated on sooner a different result would have been obtained; and I think that if an operation is undertaken before the cause of the dilatation is evident, the result in malignant cases should be as good as in those due to cicatricial contraction. In the later condition my mortalities have been nil, and I think that death under such circumstances should not occur except in a case which has been neglected for too long a time, or by some surgical accident. Under these circumstances I feel justified in strongly urging the necessity of not waiting till a clear diagnosis of malignancy has been established, but on the other hand of performing an exploratory operation in every doubtful case.

